# Adult onset Still’s disease in the elderly: a case-based literature review

**DOI:** 10.1186/s41927-021-00183-6

**Published:** 2021-04-20

**Authors:** Arash Mollaeian, Jingjing Chen, Nina N. Chan, Gregory A. Nizialek, Christopher J. Haas

**Affiliations:** 1 MedStar Health Internal Medicine Residency Program, Baltimore, MD USA; 2 Georgetown University School of Medicine, Washington, DC USA

**Keywords:** Adult onset Still’s disease, Autoinflammatory disorders, Hyperferritinemia, Arthritis, Elderly, Case report

## Abstract

**Background:**

Adult onset Still’s disease (AOSD) is a rare inflammatory disorder that classically presents with high spiking fevers, evanescent rash, and arthritis. The diagnosis is one of exclusion and can be further complicated by atypical presentations, particularly in elderly patients in whom AOSD is very rare.

**Case presentation:**

A case of AOSD in a 73-year-old woman with a non-classic presentation, leading to delayed diagnosis and management, is presented along with a review of the English literature for AOSD cases in elderly people over 70 years of age. Thirty nine case reports and series were identified and the current case was added, totaling 42 individual cases. Significant findings included a four-times higher prevalence in females, a higher prevalence of macrophage activation syndrome despite lower mortality, the presence of pruritic rash in almost one fifth of the cases, and high prevalence of delayed diagnosis.

**Conclusions:**

AOSD in the elderly may vary from the classic criteria described in the medical literature and may lead to delayed diagnosis and management. Further evaluation and better characterization of AOSD in the elderly remains an area of interest.

## Background

Adult onset Still’s disease (AOSD) is one cause of fever of unknown origin in adults and a diagnostic challenge for clinicians. Due to its rarity, the epidemiology of AOSD is not well understood, however a prevalence of 1 to 34 cases per million people has been reported [1]. While a slight female predominance exists, the overall incidence in the female and male general population seems to be equal [1, 2]. The overwhelming majority of cases present between the ages of 16–35, however 10% of cases present after 50 years of age [1–3].

AOSD in the elderly is rare and not well studied, therefore its prevalence and characteristics in this age group are not well understood. This can potentially lead to misdiagnosis or delayed diagnosis in this population, subsequently leading to delay in proper management and possible increase in complications. We present a case of AOSD in a 73-year-old woman with an atypical presentation that led to a 6-month delay in diagnosis and management. As the age of the patient was one major factor for diagnostic delay, we reviewed and analyzed the published English literature, after performing a Medline and Embase search for case reports and case series pertaining to AOSD in patients older than 70, and summarize the results.

## Case presentation

**Fig. 1:**
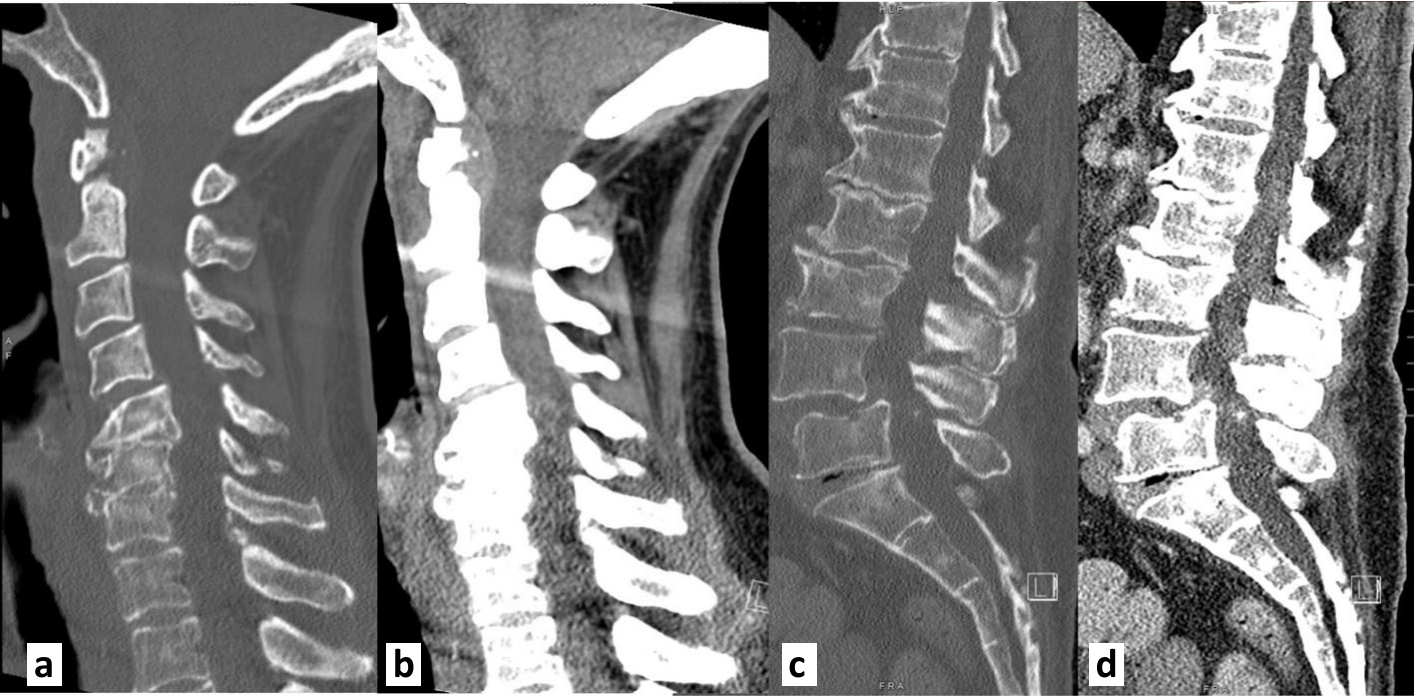
Computed tomography scan of cervical and lumbar spines. **a-b** Diffuse degenerative disc disease and spondylosis significantly at C5-C6 and C6-C7. Anterior offset of C4 on C5. **c**-**d** Degenerative disc disease and diffuse spondylotic changes at L4-L5, L5-S1 levels in lumbar spine along with disc space narrowing at all levels. Grade 1 anterolisthesis of L4 on L5

**Fig. 2:**
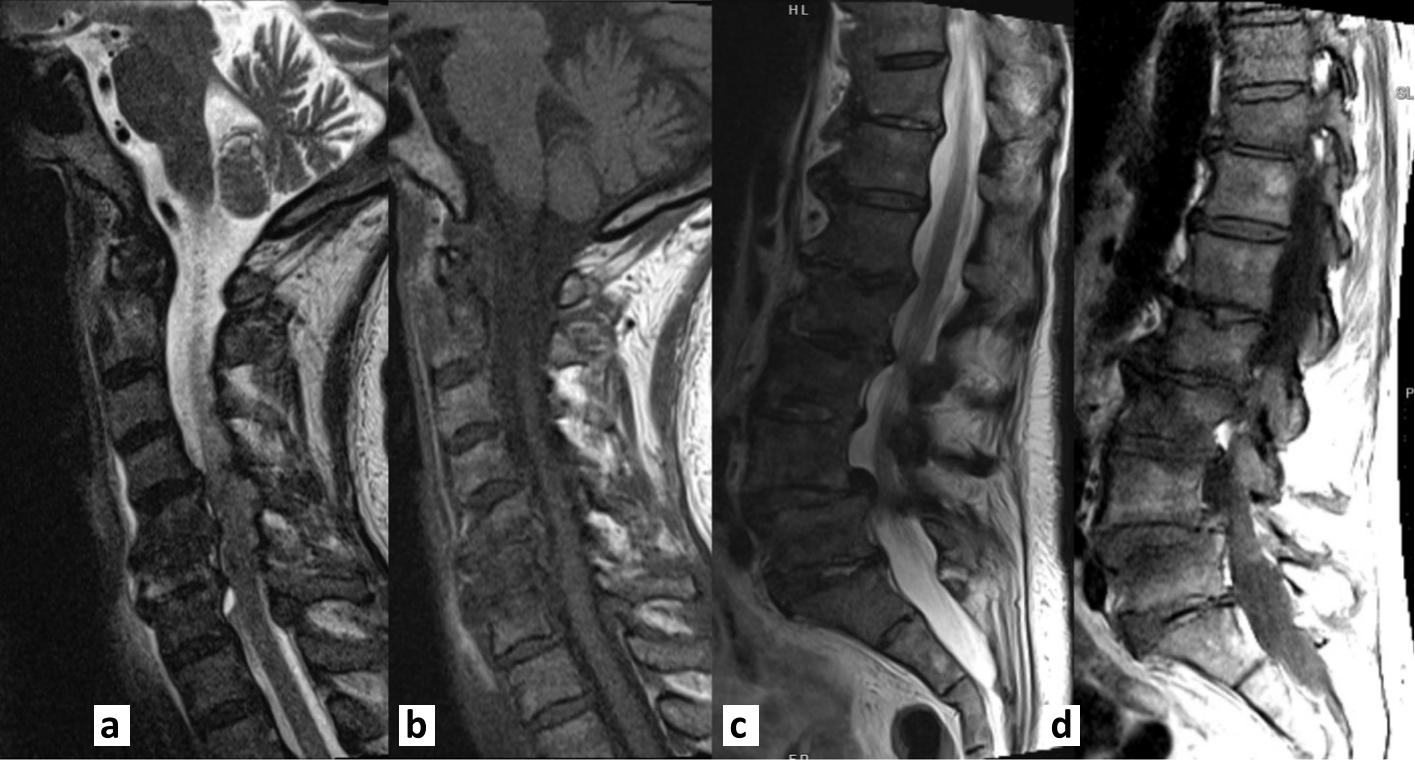
Magnetic resonance imaging of the cervical and lumbar spines. **a-b** T1 and T2 images of cervical spine showing endplate edema at C6-C7 level. Chronic degenerative changes without cord compression. **c-d** T1 and T2 images of cervical spine showing type I Modic change and enhancement along the endplates involving L2-L3 through L4–5, slightly increased at the L4-L5. Fluid signal intensity within the L3-L4 and L5-S1 disc levels with lesser involvement of L4-L5, L1-L2, and L2–3

A 73-year-old woman with past medical history of diabetes mellitus, degenerative disc disease, and osteoarthritis presented to the emergency department due to progressively worsening back pain for 1 month. The patient described the pain as intermittent, yet progressive, becoming constant, worsening with movement and lifting, and radiating to bilateral thighs without associated numbness, weakness, morning stiffness, or constitutional symptoms. She endorsed a prior history of urinary incontinence, unchanged during the current presentation. Physical exam was significant for low grade fever at 38.1^o^ C, L5-S1 spinal tenderness, and a positive bilateral straight leg raise. She had an unlimited active and passive range of motion of the bilateral lower extremities but was noted to have significant pain on knee extension and flexion, without joint swelling or erythema. Initial laboratory findings were significant for leukocytosis (20.6 k/uL), moderate microcytic hypochromic anemia, and mild transaminase elevation. Computed tomography (CT) of the lumbar spine revealed diffuse degenerative changes and spinal stenosis at L4-L5, without mass, fluid collection, fracture or dislocation (Fig. 1). Magnetic resonance imaging (MRI) of the cervical and lumbar spine was negative for cord compression or epidural abscess, however was worrisome for possible discitis/osteomyelitis at C6-C7 and L2-L5 levels (Fig. 2). The patient was started on empiric broad-spectrum antibiotics and admitted to the medical floor.

**Fig. 3:**
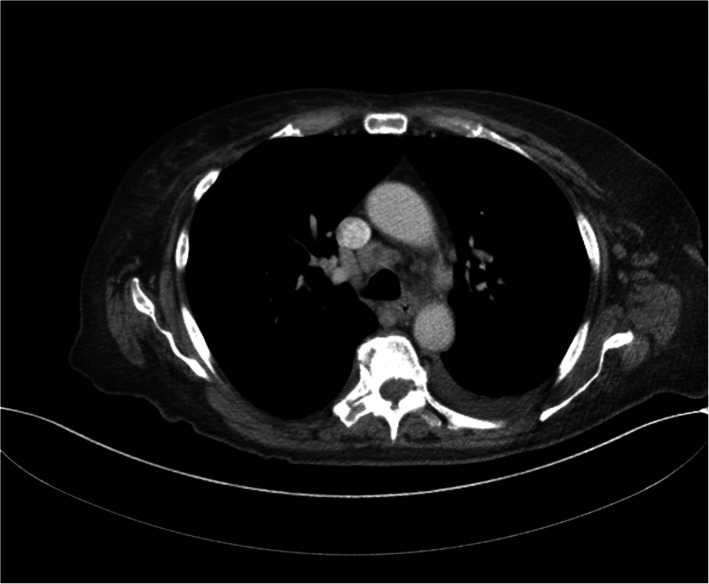
**a** Temperature and white blood cell trend during 1st and 2nd admissions and their associations with steroids administration. **b** Ferritin and CRP trend throughout the disease course and their association with different therapies

**Fig. 4:**
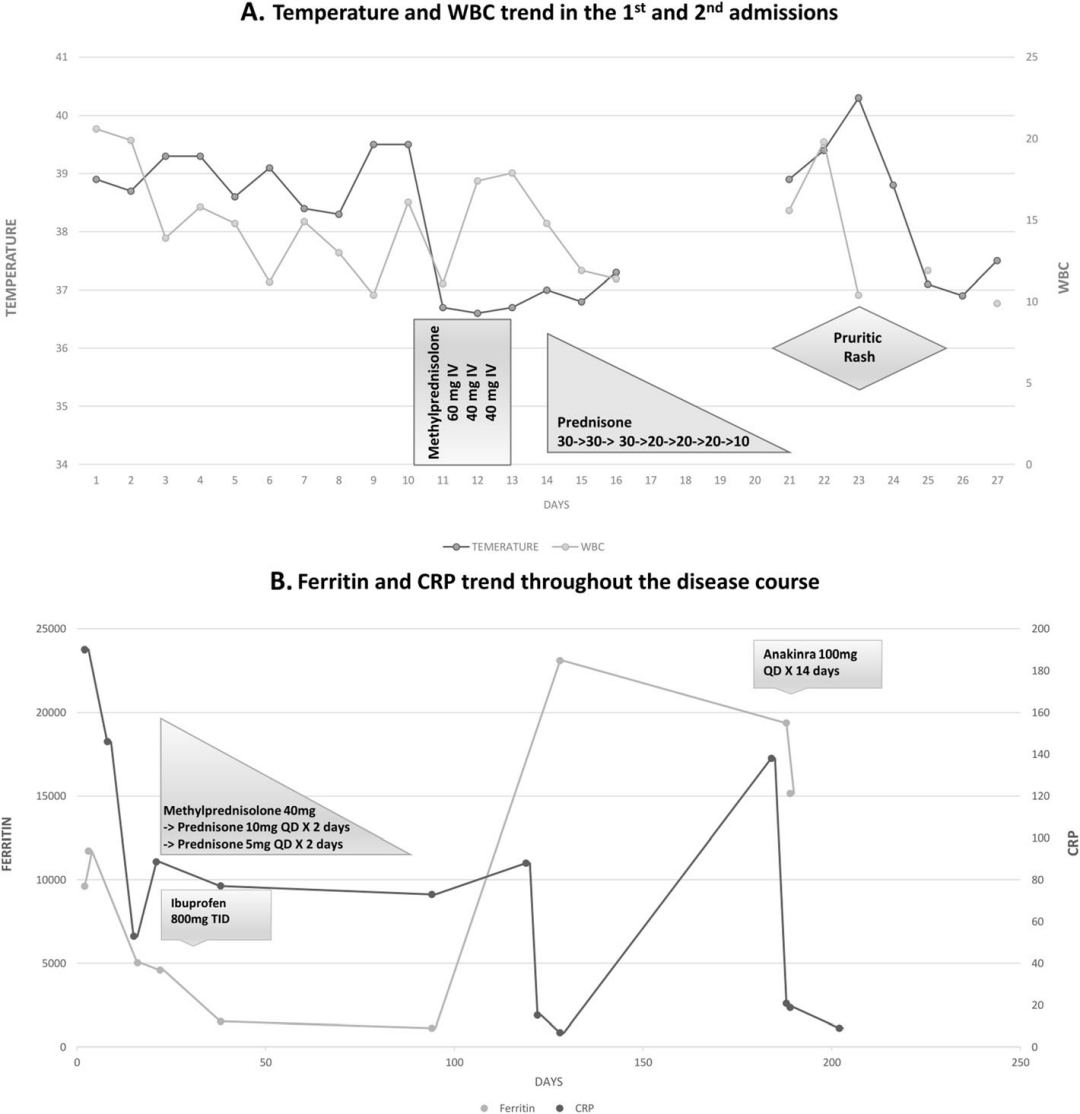
Computed tomography scan of chest. Multiple mediastinal lymph nodes are present with the largest one measuring 1.75 × 1.0 cm

Further workup revealed markedly elevated inflammatory markers, specifically a ferritin of 9617 ng/mL (Table 1). The patient continued to spike quotidian fevers up to 39 °C (Fig. 3) despite broad-spectrum antibiotics, persistently negative blood cultures, and a negative tuberculosis T-spot. CT scans of chest, abdomen and pelvis revealed a moderate left-sided pleural effusion, multiple mediastinal lymph nodes with the largest measuring 1.8 × 1.0 cm, and mild ascites (Fig. 4). CT-guided biopsy of the superior aspect of the L5 vertebrae revealed normal bone marrow and was negative for bacterial, fungal, and acid-fast bacillus organisms, ruling out infectious and non-infectious osteomyelitis. Additional diagnostic evaluation including human immunodeficiency virus (HIV), Syphilis, Brucella, Cryptococcus, Hepatitis C, and Histoplasma were all negative. Transesophageal echocardiogram to exclude culture-negative infective endocarditis was unremarkable. Bone marrow biopsy, flow cytometry, serum and urine electrophoresis were normal. Autoimmune work-up revealed a positive Anti-Nuclear Antibodies (1:160, Nucleolar staining pattern) with an elevated C4 complement, but was otherwise unremarkable (Table 1). The patient subsequently developed worsening left knee pain and swelling with limited range of motion, prompting arthrocentesis. Synovial fluid analysis demonstrated calcium pyrophosphate deposition (CPPD) and the patient was started on methylprednisolone 40 mg daily with resolution of quotidian fevers as well as improved leukocytosis. She remained afebrile on steroids and was eventually discharged to subacute rehabilitation with intravenous ceftriaxone for a planned 6–8 weeks course for possible spinal discitis/osteomyelitis (despite the noted negative culture and biopsy results) and prednisone taper for CPPD.

**Table 1:** Lab values over the course of disease and final diagnosis

Labs (Range)	Result on Day 0	Result on Day 28	Results on Day 120	Results on Day 180
WBC (4–10.8 k/uL)	20.6	15.6	15	14.5
Neutrophil (43–75%)	91.9	92.4	86.6	94
Lymphocyte (15–45%)	4	4.5	8.5	2.8
Eosinophil (0–6%)	0	0.3	0.1	0.1
Hgb (11–14.5 g/dL)	7	7.7	8.9	7.4
Platelet (145–400 k/uL)	402	243	321	303
ESR (0–22 mm/hr)	117	60	56	91
CRP (0–3 mg/L)	> 190	88.5	15.2	18.8
Ferritin (5–148 ng/mL)	9617	4585	23,096	15,141
AST (3–34 units/L)	58	18	41	41
ALT (15–41 units/L)	47	14	10	6
ANA (< 1:40)	1:160		Negative	
C3 (90–180 mg/dL)	142	110	90	
C4 (10–40 mg/dL)	40.2	33	24.2	
RF (0–15 IU/mL)	< 10		8	
Anti-CCP (0–19 Units)	11			
ds-DNA Ab		Negative		
Anti-Smith Ab		Negative		
ANCA	Negative		Negative	
Anti-Ro (SSA)	Negative		Negative	
Anti-La (SSB)	Negative		Negative	
ACA IgG	Negative			
ACA IgM	Negative			
IgA (70–400 mg/dL)	434		458	
IgG (700–1600 mg/dL)	676		776	
IgG4 (1–123 mg/dL)	57			
IgM			195	
Free Kappa (3.3–19.4 mg/L)	85.35		36	
Free Lambda (5.71–26.3 mg/L)	58.33		52	
K:L (0.26–1.65)	1.46		0.69	
IL-2 Receptor			10,000 pg/mL	
Haptoglobin (30–200 mg/dL)	515			
Absolute Reticulocyte (0.02–0.1 million/μl)	0.043			
Vitamin B9 (4.1–55.4 ng/mL)	15.6			12.9
Vitamin B12 (211–911 pg/mL)	422			807
Iron (50–170 mcg/dL)	9		17	
TIBC (265–497 mcg/dL)	115		195	
Iron Sat (15–50%)	8		9	

*WBC* White Blood Cell, *Hgb* Hemoglobin, *Ab* Antibody, *Ig* Immunoglobulin, *ESR* Erythrocyte Sedimentation Rate, *CRP* C-Reactive Protein, *ANA* Anti-Neutrophil Antibody, *ANCA* Anti-Neutrophil Cytoplasmic Antibody, *C3* Complement 3, *C4* Complement 4, *ds-DNA* Double-Stranded DNA, *RF* Rheumatoid Factor, *CCP* Cyclic Citrulinated Peptide, *SS-A* Sjӧgren-Syndrome-related-antigen A, *SS-B* Sjӧgren-syndrome-related-antigen B, *ACA* Anti Cardiolipin Ab, *AST* Aspartate Aminotransferase, *ALT* Alanine Aminotransferase, *IL* Interleukin, *TIBC* Total Iron Biding Capacity, *Sat* Saturation

**Fig. 5:**
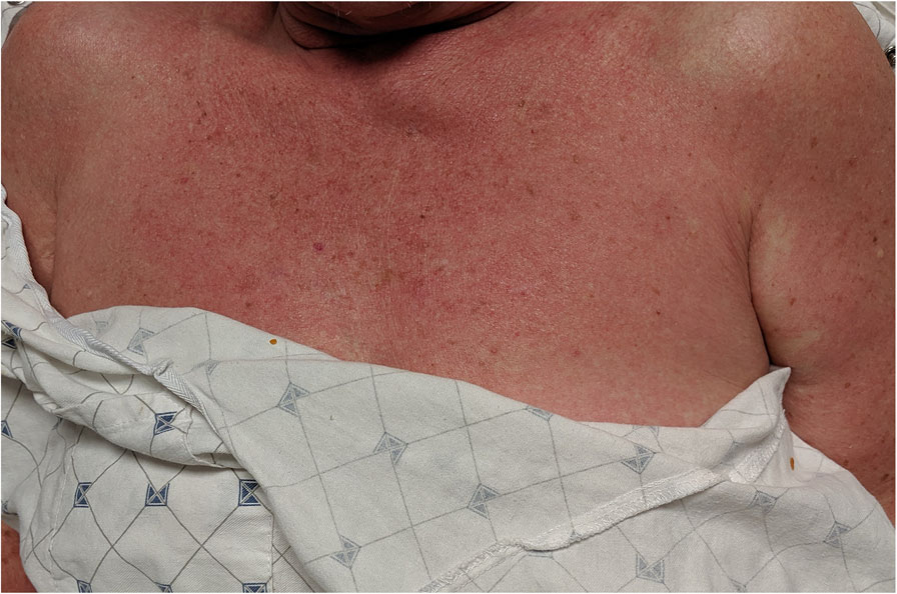
Pruritic confluent macular rash involving the chest and back

Five days after discharge, she developed spiking fevers associated with a diffuse pruritic rash involving the anterior chest and neck and was readmitted to our hospital. No significant changes were noted except for a rapid taper of Prednisone to 10 mg. Physical exam was significant for a fever of 40.3 °C and a macular, pink truncal rash. Repeat laboratory findings demonstrated persistent leukocytosis (15.6 k/uL) with neutrophilic predominance (92.4%) and persistently elevated inflammatory markers. Blood cultures were obtained and empiric broad-spectrum antibiotics were initiated. A single dose of methylprednisolone 40 mg IV was administered, leading to fever resolution and improvement of the pruritic rash. Repeat CT scan demonstrated persistent mildly enlarged mediastinal lymph nodes, resolution of the previously observed pleural effusion and ascites,, and no organomegaly. Her antibiotics were switched to meropenem due to concern for osteomyelitis and ultimately discontinued in light of persistently negative blood cultures. Twenty-four hours after discontinuing Meropenem, a time that coincided with tapering of prednisone to 10 mg, she developed fever and a severe, pruritic rash involving the entire trunk (Fig. 5) that disappeared with resolution of fever. Mediastinal lymph node biopsy was deferred due to the high risk of complications. She remained afebrile, and in discussion with Rheumatology, prednisone was tapered to 5 mg, leading to recurrence of fever and the previously noted pruritic rash. Additional diagnostic workup demonstrated negative double stranded DNA antibody, anti-Smith antibody, and normal serum angiotensin converting enzyme. Given the negative workup and fulfillment of Yamaguchi criteria (Table 2), the patient was diagnosed with Adult Onset Still’s Disease. She was started on Ibuprofen 800 mg three times a day and prednisone tapered to 5 mg for 2 days and then discontinued. The patient remained asymptomatic, afebrile and demonstrated improvement in the rash. She was discharged home and instructed to follow-up as outpatient with rheumatology.

**Table 2:** Yamaguchi’s and Fautrel’s criteria met by patient

Yamaguchi’s criteria [4] At least 5 criteria including 2 major criteria	Criteria met by our case	Fautrel’s criteria [5] 4 major or 3 major and 2 minor criteria	Criteria met by our case
Major:		**Major:**	
• Fever > 39 °C, lasting 1 week or longer • Arthralgia or arthritis, lasting 2 weeks or longer • Typical rash • Leukocytosis > 10,000/mm3 with > 80% polymorphonuclear cells	+ + -^a^ +	**•** Spiking fever ≥39 °C **•** Arthralgia **•** Transient erythema **•** Pharyngitis **•** Polymorphonuclear cells ≥80% **•** Glycosylated ferritin ≤20%	+ + +^a^ - +
Minor:		**Minor:**	
• Sore throat • Lymphadenopathy • Hepatomegaly or splenomegaly • Abnormal liver function tests • Negative tests for antinuclear antibody and rheumatoid factor	- + - + −/+	**•** Maculopapular rash **•** Leukocytosis ≥10,000/mm3	+ +
Exclusion:			
• Infections • Malignancies (mainly malignant lymphoma) • Other rheumatic disease (mainly systemic vasculitides)	+ + +		

^a^The evanescent rash in our case presented on 2nd admission after and was pruritic, therefore not meeting the Yamaguchi’s criteria

Three months later her quotidian fevers and arthralgia recurred and she was admitted to a neighboring medical center. She was evaluated by multiple sub-specialty services, but extensive infectious, autoimmune, and hematological work-up remained negative. She was empirically started on colchicine with some improvement but continued to spike quotidian fevers. Positron Emission Tomography scan showed hilar and supraclavicular lymphadenopathy. An ultrasound-guided biopsy of supraclavicular nodes showed reactive lymphoid tissue without granulomas. She was discharged without a definitive diagnosis and a month later underwent bilateral temporal artery biopsy that was negative for giant cell arteritis. Over the course of the next 2 months she suffered from relapsing symptoms of fever and arthralgia, prompting readmission to the neighboring hospital. Comprehensive evaluation was once again negative except for elevated inflammatory markers (Table 1). Rheumatology re-evaluated the patient and diagnosed her with AOSD. The patient was started on Anakinra 100 mg daily and remained asymptomatic with normal inflammatory markers on outpatient follow-up (Fig. 3).

## Discussion and conclusions

**Fig. 6:**
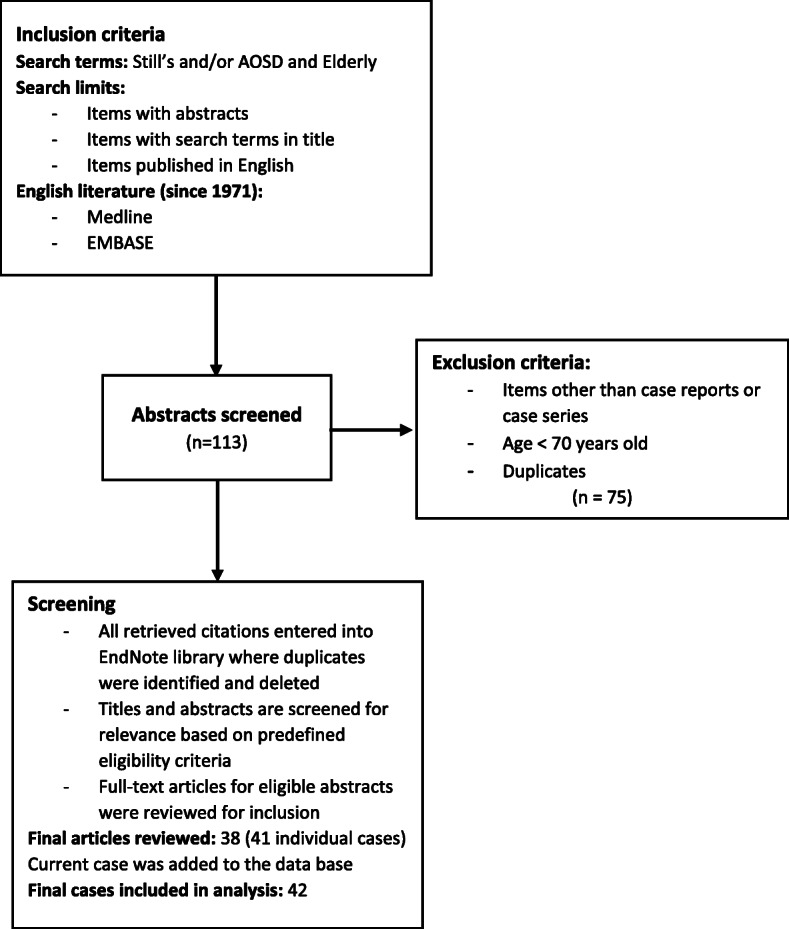
Literature review flow chart

We performed a web-based search in Medline and Embase databases of English language medical literature using the key terms “Still’s”, “AOSD” and “elderly”, and filtered the search for age 70 years and older. A total of 113 articles were identified. Duplicates and articles other than case reports and series were excluded from the initial pool of articles. Thirty eight case reports and series (e.g. abstracts) [4–41] with a total of 41 individual cases remained and the present case was added to the database, making for a total of 42 individual cases (Fig. 6). We then retrospectively reviewed each case with attention to its demographics (such as gender, age), presenting signs and symptoms, laboratory findings, complications such as macrophage activation syndrome (MAS), time to diagnosis and finally treatment regimens. Collected data were categorized and using SAS University edition, the identified data were analyzed as median ± interquartile range (IQR) or percentage, where appropriate. To compare the patients who developed MAS and those who did not, we further performed Chi-square/Fisher test and Wilcoxon rank sum test to compare the categorical variables and continuous variables, respectively. Logistic regression analysis was used to analyze factors, including leukocytosis and ferritin level that independently predict MAS. Two-sided hypothesis testing was utilized to run statistical analysis and a *p* value of < 0.05 was considered statistically significant. All analytic operations were performed using SAS university edition.

The mean age of onset was 76.7 years among all 42 cases. Thirty four cases were reported in women with a female to male ratio of 4 to 1. The most common presenting symptom was fever (100%), with quotidian fever as the predominant pattern. The next most common symptoms on presentation were arthralgia (90.48%) and rash (80.95%) (Table 3). Pruritic rash was reported in 21% of cases with rash. Other less frequently reported features were sore throat (52.38%), hepatosplenomegaly (33.33%), and serositis (16.67%). Macrophage Activation Syndrome (MAS) was reported in nearly 24% of cases without an associated increase in mortality. Leukocytosis was the most prevalent lab finding (78.37%), with a median count of 13,700 (k/uL) and was found to be negatively associated with MAS (*p* = 0.04). Ferritin level greater than 1000 ng/ml was significantly associated with MAS (*p* = 0.01). Elevation of transaminases was present in two thirds of cases but was not associated with MAS. The most common treatment modality was steroids, followed by conventional disease modifying anti-rheumatic drugs (DMARDs), non-steroidal anti-inflammatory drugs (NSAIDs), biologic DMARDs, and miscellaneous therapies, respectively (Table 3). Delayed diagnosis was defined as diagnosis made later than 1 month with a prevalence as high as 90%.

**Table 3:** Demographic and clinical characteristics of adult onset Still’s disease in elderly cases [4–41]

Clinical Characteristics	Total (N, %)^**a**^	Patients with MAS^**b**^ (N, row %)	Patients without MAS (N, row %)	***p***-value*
Patient count	42 (100%)	10 (23.81%)	32 (76.19%)	
AGE (YEAR), (MEDIAN, IQR)	75, 7	74.5, 8	75, 6	0.49
Female	34 (80.95%)	10 (23.81%)	24 (57.14%)	0.16
Fever	42 (100%)	10 (23.81%)	32 76.19%)	1.00
Rash	34 (80.95%)	10 (23.81%)	24 (57.14%)	0.16
Pruritic rash	9 (21.43%)	4 (9.52%)	5 (11.90%)	0.23
Arthralgia/Myalgia	38 (90.48%)	7 (16.67%)	31 (73.81%)	0.04
Serositis	7 (16.67%)	1 (2.38%)	6 (14.29%)	0.49
Hepatosplenomegaly	14 (33.33%)	3 (7.14%)	11 26.19%)	0.06
Sore throat	22 (52.38%)	6 (14.29%)	16 (38.10%)	0.31
Leukocytosis, (Median, IQR)	13,700, 8800	9000, 5895	16,000, 9470	< 0.01
Hyperferritinemia, (Median ± IQR)	5336, 15,447	41,932, 70,250	2684, 7821	< 0.01
Transaminitis	28 (66.67%)	7 (16.67%)	21 (50.0%)	1.00
Delayed Diagnosis (> 1 month)	38 (90.48%)	9 (21.43%)	29 (69.05%)	< 0.01
Corticosteroids	39 (92.36%)	9 (21.43%)	30 (71.43%)	0.28
NSAIDs^c^	14 (33.33%)	1 (2.38%)	13 (30.95%)	0.04
Conventional DMARDs^d^	17 (40.48%)	5 (11.90%)	12 (28.57%)	0.15
Biologic DMARDs	8 (19.05%)	3 (7.14%)	5 (11.90%)	0.09
Miscellaneous Treatment	8 (19.05%)	3 (7.14%)	5 (11.90%)	0.09

^a^ All percentages are based on the overall patients in total (42 patients)

^b^ *MAS* Macrophage activation syndrome

^c^ *NSAIDs* Nonsteroidal anti-inflammatory drugs

^c^ *DMARDs* Disease-modifying anti-rheumatic drugs

* Wilcoxon rank sum test, Chi-square test, or Fisher’s exact test was applied where it is appropriate. Statistical tests used two-sided hypothesis testing

While patients with AOSD typically present with quotidian fever, arthralgia/arthritis, and an evanescent rash [1], a variety of atypical presentations exist. In this case, we describe late onset AOSD in an elderly female who presented outside the classic age distribution with additional atypical features including a pruritic, maculopapular rash. She was noted to have additional findings of cervical and lumbar spinal discitis and CPPD arthropathy, altogether contributing to a diagnostic delay. While CPPD can rarely present with axial skeletal involvement and constitutional symptoms such as fever, the fever pattern and other accompanying features such as lymphadenopathy and rash makes CPPD unlikely to be the primary underlying pathology. Genetic evaluations was not available in this case however we believe that diseases of genetic mutation like tumor necrosis factor receptor-associated periodic syndrome (TRAPS) and the recently described VEXAS (vacuoles, E1 enzyme, X-linked, autoinflammatory, somatic) syndrome [42] could be evaluated with genetic testing in appropriate settings. Other autoinflammatory and autoimmune etiologies such as Castleman’s syndrome,, necrotizing vasculitis, relapsing polychondritis, Schnitzler’s syndrome, and drug reaction with eosinophilia and systemic symptoms (DRESS) syndrome were excluded based on absence of weight loss, hepatomegaly, splenomegaly, thrombocytopenia, eosinophilia, pancytopenia, unremarkable serum and urine electrophoreses, autoimmune workup, bone marrow, lymph node, and temporal artery biopsies.

Our literature review is limited due to small size and retrospective nature, as well as limitations in regards to patients’ survival and outcome. Nevertheless, it highlights not only AOSD prevalence but characterizes the associated symptoms and laboratory presentations in the elderly. While AOSD in the elderly shares the most common features of AOSD in general population, a variety of key differences exist in the elderly versus classic AOSD populations. We identified a 4 to 1 female to male ratio, which contrasts with the reported distribution of AOSD in general population where males and females are equally affected [1, 2]. Although it may simply be a result of an overall longer life expectancy in women compared to men, it is significant enough to be further investigated in future studies. Another notable finding in our review is the delayed diagnosis, defined as diagnosis made later than 1 month from initial presentation. Whether the delayed diagnosis is limited to elderly population, given their associated multiple comorbidities, is unclear, however it is possible that diagnostic delay may also exist in the younger population. We propose that the delayed diagnosis is likely related to the complicated nature and presentation of AOSD as well as divergence from classic diagnostic criteria in the elderly population.

The characteristic rash of AOSD is an evanescent, maculopapular pink rash that appears during febrile episodes on the extremities, trunk, and neck. Although Yamaguchi’s criteria characterize the rash as nonpruritic [43], pruritic rash and atypical skin manifestations have been reported [44] and were also present in 21% of elderly AOSD cases in our review. This finding calls for attention as it diverges from the classic non-pruritic rash of Yamaguchi criteria, which is the most widely universally used diagnostic criteria by clinicians. This can potentially lead to missed cases of AOSD whom present with atypical symptoms such as pruritic rash, delaying diagnosis and treatment.

While MAS was noted in 24% of elderly AOSD cases in our review, there was minimal mortality, in contrast to the noted mortality associated in younger populations [45, 46]. Although this discrepancy can be a result of limitation of our review in assessing the survival of each case, hypothetically it could be attributed to immune system senescence in elderly population [47] and mandates further investigations. In a recent epidemiologic study of AOSD patients from a nationwide inpatient sample database, the prevalence of MAS, disseminated intravascular coagulopathy (DIC), and thrombotic thrombocytopenic purpura (TTP) was reported to be 1.7, 1.1, and 0.4% respectively with overall inpatient mortality of 2.6%, with the highest mortality in cases of DIC [45]. The negative association of leukocytosis with MAS in our analysis, is well established in MAS cases, however this is limited in our analysis as timing of leukocyte count in regard to MAS could not be evaluated due to limitations of the case reports.

Hyperferritinemia is a common laboratory finding seen in up to 70% of AOSD cases [1, 2]. Elevation of the ferritin level more than 5 times the upper limit of normal is considered to be suggestive of AOSD [48], however isolated ferritin elevations have a low positive predictive value with a specificity as low as 46% [49]. Ferritin is considered not only to be a diagnostic marker, but also a marker of disease severity and is used to monitor response to therapy [1, 2]. Ferritin levels greater than 5000 ng/ml are considered to be suggestive of MAS or reactive hemophagocytic syndrome and are reported to occur in up to 15% of cases of AOSD [50]. In our review, ferritin greater than 1000 ng/ml was statistically associated with MAS with a *p*-value less than 0.01.

Of note in a very recent assessment of Japanese elderly AOSD patient population [50], authors reported similar findings to our review with female predominance, atypical skin lesions, and higher prevalence of complications such as MAS. A reduced survival rate was reported in that study especially in the setting of complications such as DIC and MAS, however, these findings along with findings of our literature review underscore the need for better characterization of AOSD in the elderly.

The complicated course of the present case and results of our literature review highlight the prevalence of atypical presentations of AOSD in the elderly. Notable distinguishing features include a female predominance, a pruritic rash, a mildly increased evidence of MAS, and a lower MAS-associated mortality rate in this age group. Thus, we conclude that old age and pruritic rash should not be considered exclusionary for AOSD and that current available criteria, although helpful, are not absolute, especially in cases with atypical features. This review, although limited due to small sample size, retrospective nature and lack of a comparison group, emphasizes the need for further investigations to identify potential disease markers and development of alternative diagnostic criteria for those at extremes of age and potentially a unifying and globally accepted diagnostic criteria for AOSD.

## Data Availability

Not applicable.

## Abbreviations

AOSD: Adult onset Still’s disease
CT: Computed tomography
MRI: Magnetic resonance imaging
HIV: Human immunodeficiency virus
CPPD: Calcium pyrophosphate deposition
MAS: Macrophage activation syndrome
IQR: Interquartile range
DMARDs: Disease modifying anti-rheumatic drugs
NSAIDs: Non-steroidal anti-inflammatory drugs
TRAPS: Tumor necrosis factor receptor-associated periodic syndrome
DRESS: Drug reaction with eosinophilia and systemic symptoms
DIC: Disseminated intravascular coagulopathy
TTP: Thrombotic thrombocytopenic purpura
VEXAS: Vacuoles, E1 enzyme, X-linked, autoinflammatory, somatic
