# Correction to: Adult onset Still’s disease in the elderly: a case-based literature review

**DOI:** 10.1186/s41927-021-00201-7

**Published:** 2021-05-18

**Authors:** Arash Mollaeian, Jingjing Chen, Nina N. Chan, Gregory A. Nizialek, Christopher J. Haas

**Affiliations:** 1 MedStar Health Internal Medicine Residency Program, Baltimore, MD USA; 2 Georgetown University School of Medicine, Washington, DC USA

**Correction to: BMC Rheumatol 5, 12 (2021)**

**https://doi.org/10.1186/s41927-021-00183-6**

Following publication of the original article [1], it was reported that the images used for Fig. 3 and Fig. 4 were switched. The correct figures are given in this Correction article and the original article has been corrected.

**Fig. 3:**
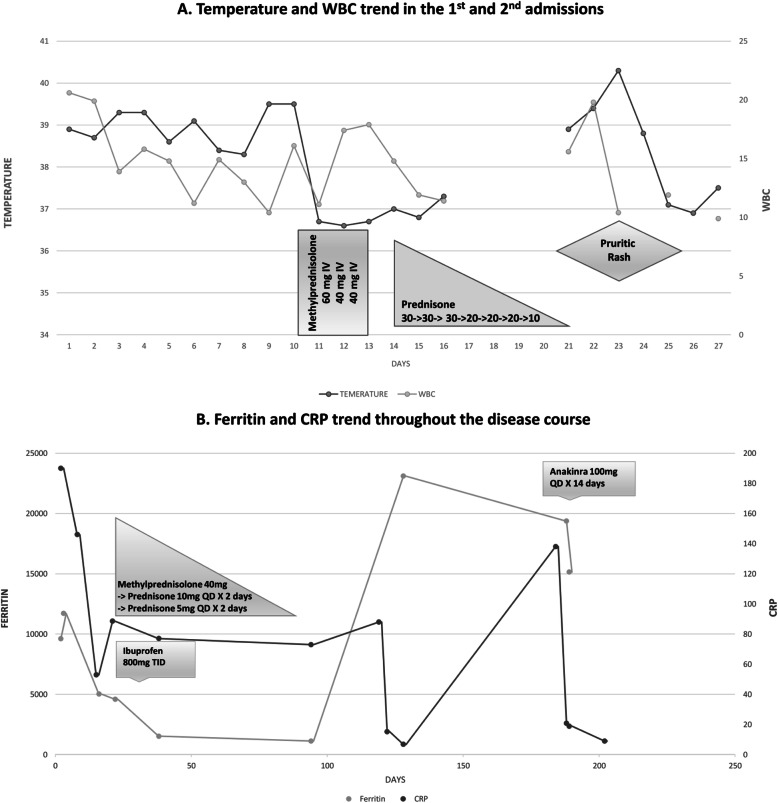
**a** Temperature and white blood cell trend during 1st and 2nd admissions and their associations with steroids administration. **b** Ferritin and CRP trend throughout the disease course and their association with different therapies

**Fig. 4:**
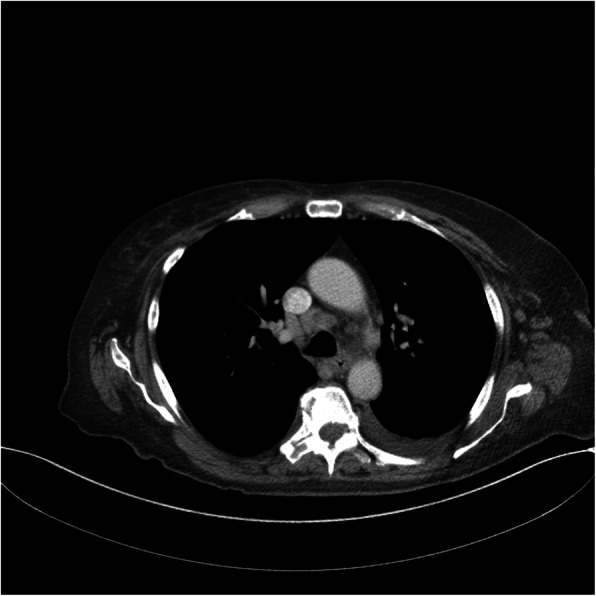
Computed tomography scan of chest. Multiple mediastinal lymph nodes are present with the largest one measuring 1.75 × 1.0 cm
